# *QuickStats:* Percentage of Women Who Smoked* Cigarettes During Pregnancy, by Race and Hispanic Origin^†^ — National Vital Statistics System, United States, 2016 and 2022

**DOI:** 10.15585/mmwr.mm7250a5

**Published:** 2023-12-15

**Figure:**
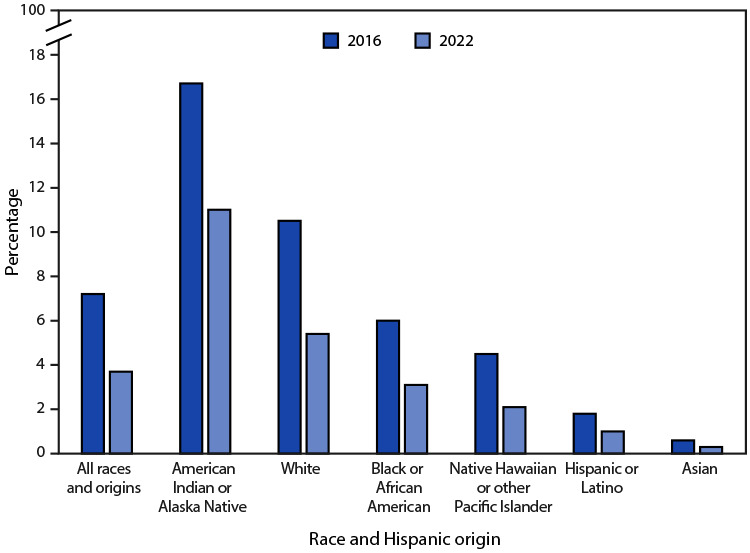
The percentage of women who smoked cigarettes at any time during pregnancy declined from 7.2% to 3.7% from 2016 to 2022. Smoking during pregnancy declined in each race and Hispanic-origin group during this period. Percentages declined from 16.7% to 11.0% among non-Hispanic American Indian or Alaska Native women, from 10.5% to 5.4% among non-Hispanic White women, from 6.0% to 3.1% among non-Hispanic Black or African American women, from 4.5% to 2.1% among non-Hispanic Native Hawaiian or other Pacific Islander women, from 1.8% to 1.0% among Hispanic or Latino women, and from 0.6% to 0.3% among non-Hispanic Asian women.

**Source:** National Center for Health Statistics, National Vital Statistics System, Natality Data File, 2022. https://www.cdc.gov/nchs/nvss/births.htm

For more information on this topic, CDC recommends the following link: https://www.cdc.gov/tobacco/basic_information/health_effects/pregnancy/

